# Supplementary figures and images for: Repressing miR-23a promotes the transdifferentiation of pancreatic α cells to β cells via negatively regulating the expression of SDF-1α
Source: PLoS One. 2024 Mar 22;19(3):e0299821. doi: 10.1371/journal.pone.0299821 (PMC10959391; doi:10.1371/journal.pone.0299821)

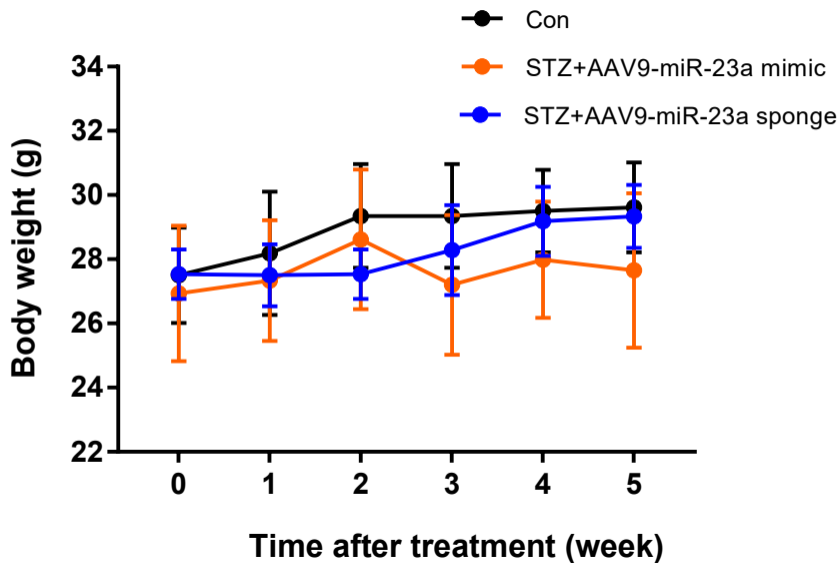

Supplement: S1 Fig — (PDF) [file pone.0299821.s001.pdf]

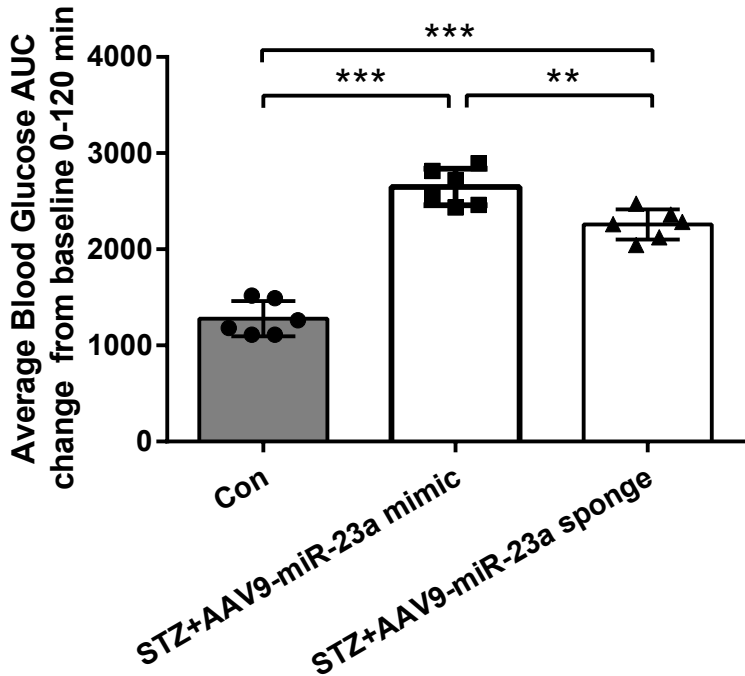

Supplement: S2 Fig — (PDF) [file pone.0299821.s002.pdf]

**Fig 1J**

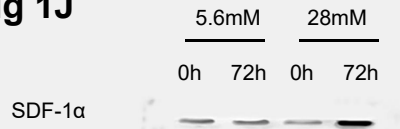

**Fig 1J**

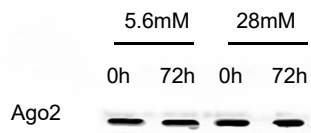

**Fig 2B**

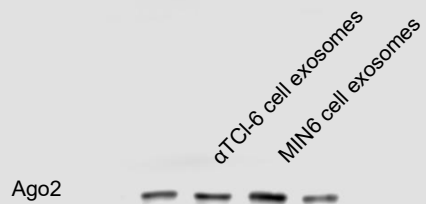

**Fig 2B**

TSG101

αTCL-6 cell exosomes  
MIN6 cell exosomes

**Fig 2B**

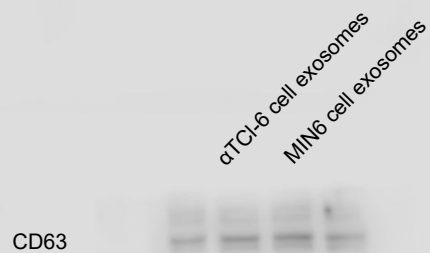

Fig 2E

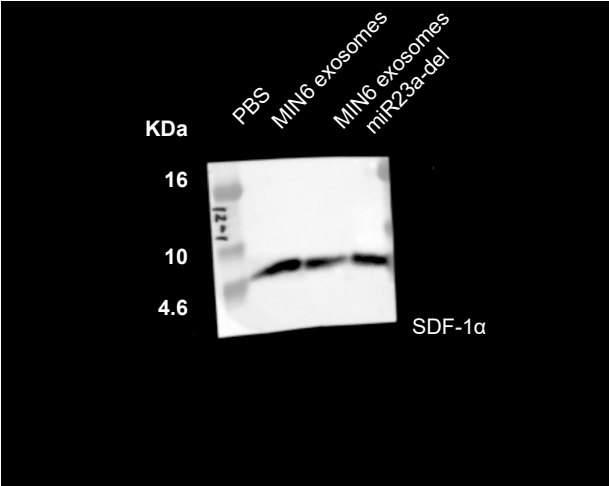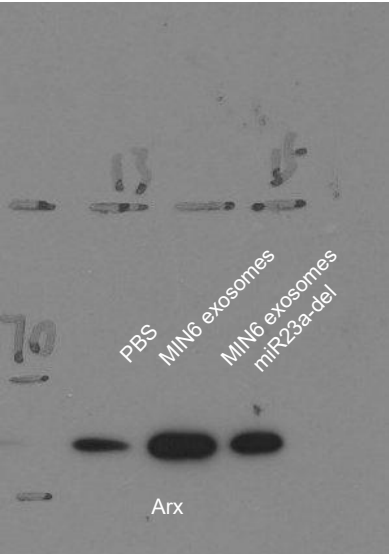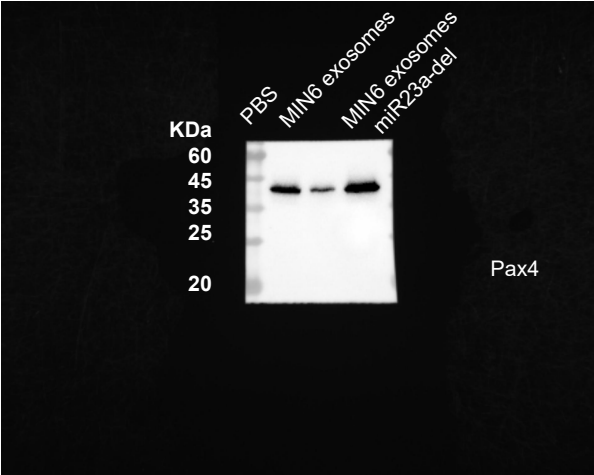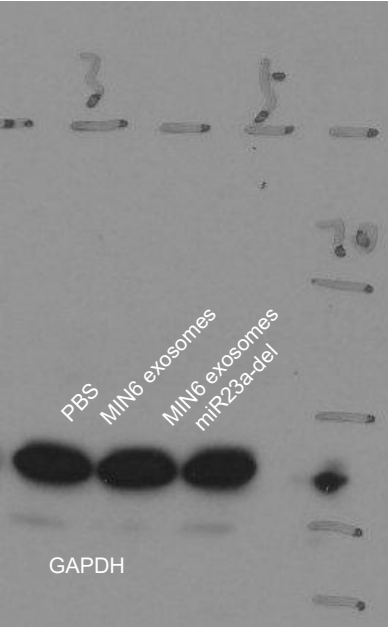

Fig 2F

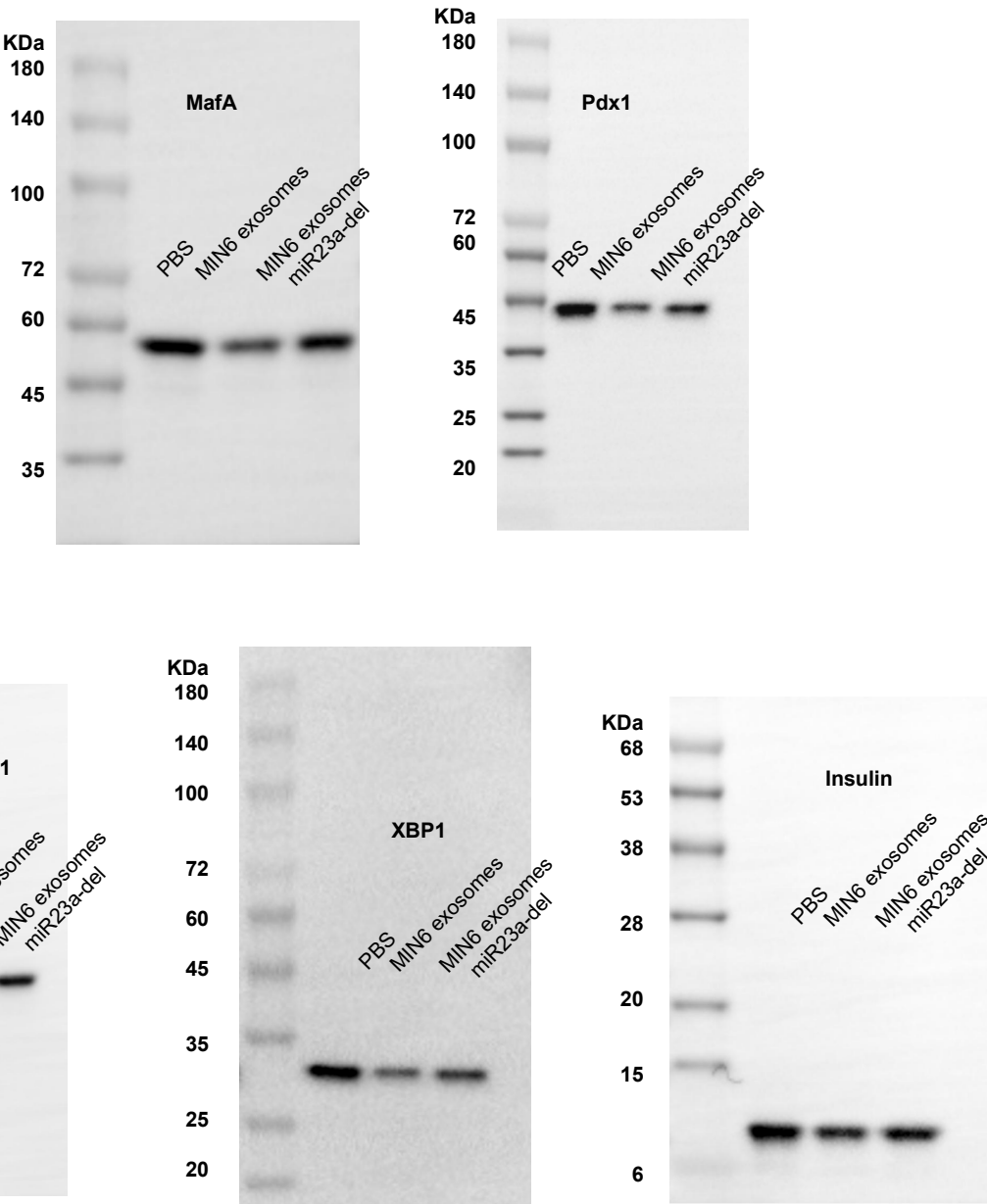

Fig 2F

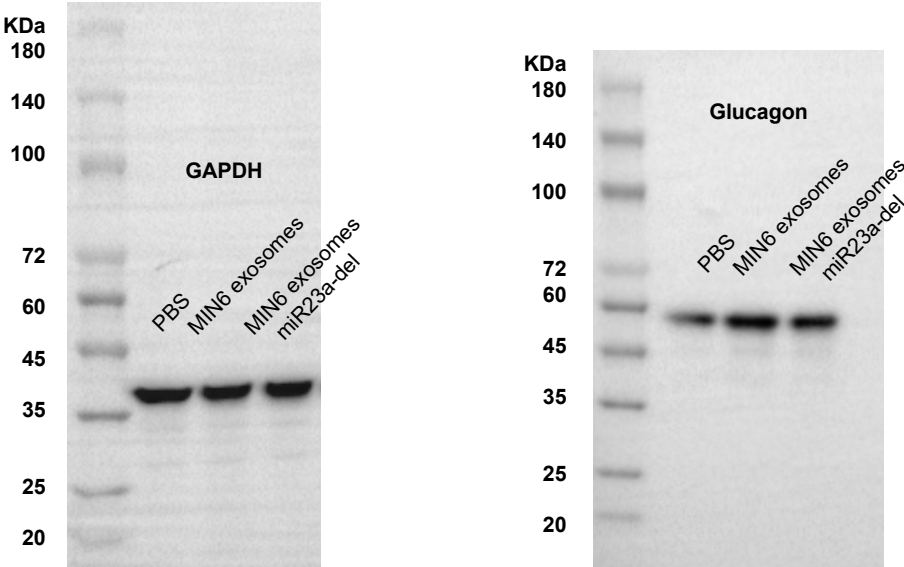

Fig 3D

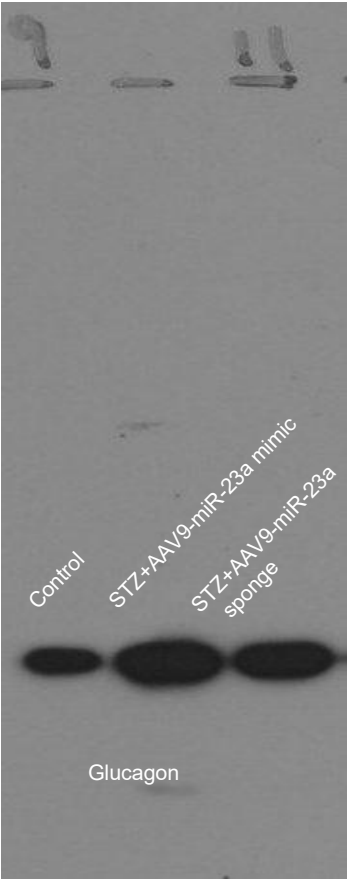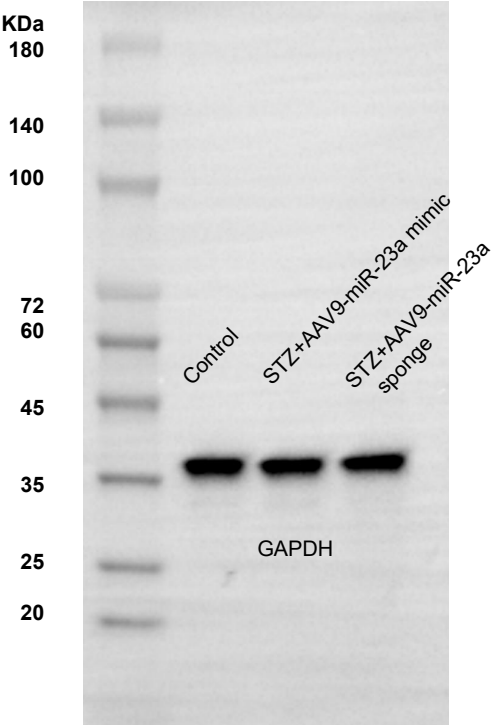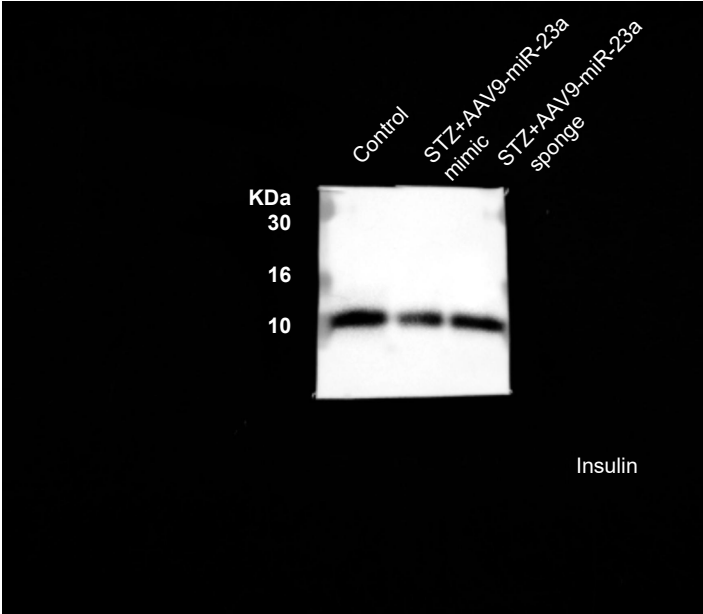

**Fig 3G**

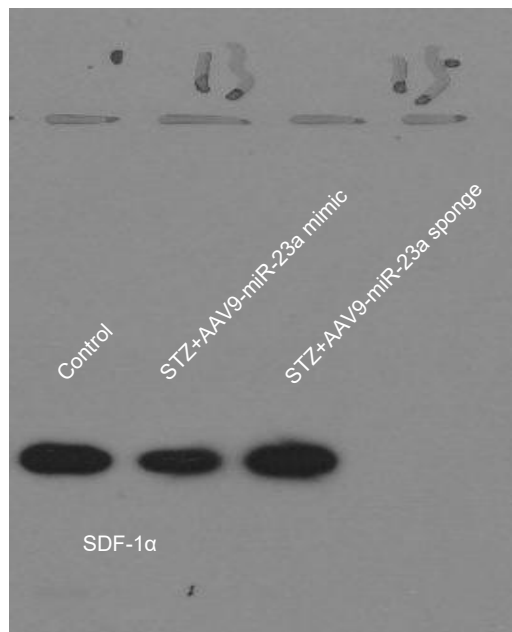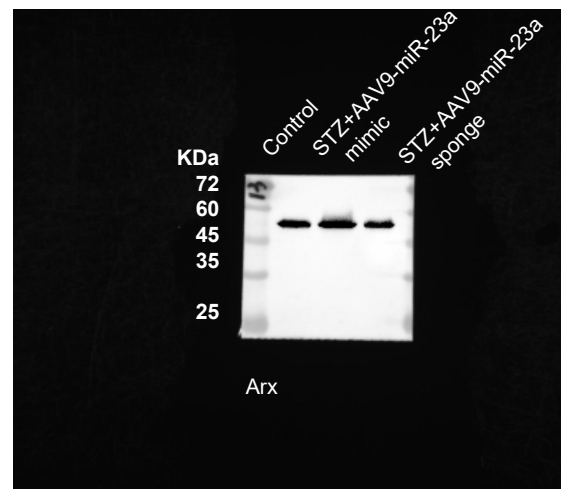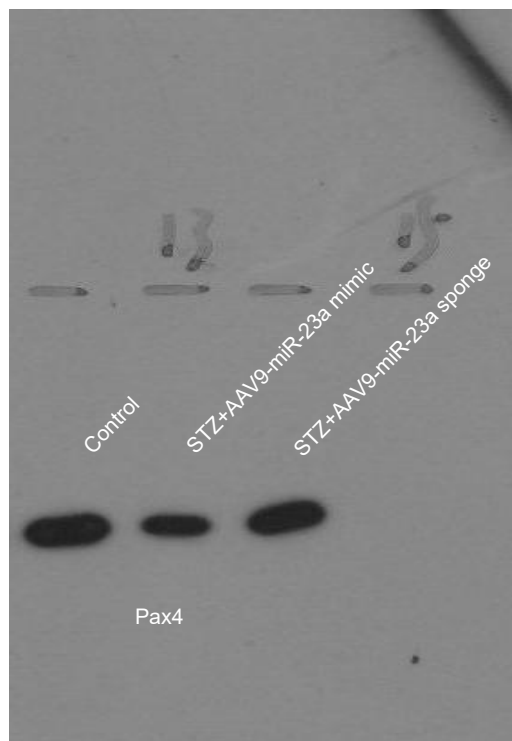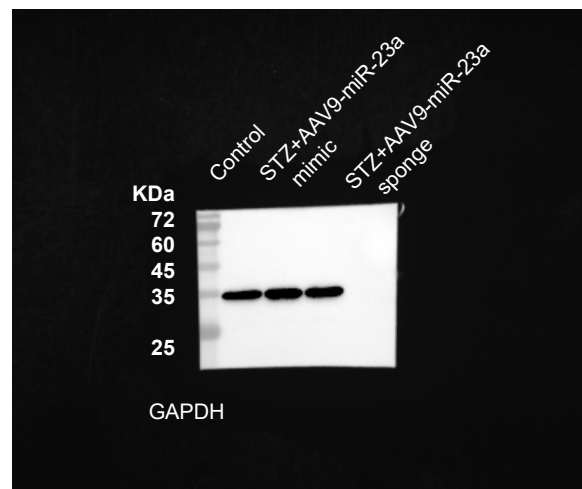

Supplement: S3 Fig — (PDF) [file pone.0299821.s003.pdf]
