# Supplementary material for: Repressing miR-23a promotes the transdifferentiation of pancreatic α cells to β cells via negatively regulating the expression of SDF-1α
Source: PLoS One. 2024 Mar 22;19(3):e0299821. doi: 10.1371/journal.pone.0299821 (PMC10959391; doi:10.1371/journal.pone.0299821)
Supplement: S1 Table — (DOCX) [file pone.0299821.s004.docx]

Supplementary Table. Sequences of Primers

| Gene name |  | 5’-3’Sequence |
| --- | --- | --- |
| **GAPDH** | Forward | AGGTCGGTGTGAACGGATTTG |
|  | Reverse | GGGGTCGTTGATGGCAACA |
| **U6** | Forward | CTCGCTTCGGCAGCACA |
|  | Reverse | AACGCTTCACGAATTTGCGT |
| **miR-23-3p** | Forward | ACCGTATCACATTGCCAGGG |
|  | Reverse | GTGCAGGGTCCGAGGT |
| **SDF1** | Forward | TGCATCAGTGACGGTAAACCA |
|  | Reverse | TTCTTCAGCCGTGCAACAATC |
| **Arx** | Forward | GGCCGGAGTGCAAGAGTAAAT |
|  | Reverse | TCGATGCAGTAGGAGGAGAGC |
| **Pax4** | Forward | AGGGGGACTCTTTGTGAATGG |
|  | Reverse | ACCTGTGCGGTAGTAGCGT |
| **ALDH1A3** | Forward | AGGCTGTATTAAGACCTTCAG |
|  | Reverse | GGAAGTTCCATGGTGTAATG |
| **Neurog3** | Forward | CTAAGAGCGAGTTGGCACTGA |
|  | Reverse | GAGGTTGTGCATTCGATTGCG |
| **Insulin** | Forward | TCTTCTACACACCCATGTCCC |
|  | Reverse | GGTGCAGCACTGATCTAC |
| **MafA** | Forward | GGCGCACGCTCAAGAACC |
|  | Reverse | ATGTGCCGCTGCTGCACC |
| **Pdx1** | Forward | CGGACATCTCCCCATACG |
|  | Reverse | AAAGGGAGCTGGACGCGG |
| **Neurod1** | Forward | ACTCCAAGACCCAGAAACTGTC |
|  | Reverse | ACTGGTAGGAGTAGGGATGCAC |
| **Ucn 3** | Forward | GAGGGAAGTCCACTCTCGGG |
|  | Reverse | TGTTGAGGCAGCTGAAGATGG |
| **Ero1-beta** | Forward | TGGAGTTCTGGATGATTGCTT |
|  | Reverse | TCTTCTGCCCAGAAAGGACA |
| **XBP1** | Forward | GCAGCAAGTGGTGGATTTGG |
|  | Reverse | AGATGTTCTGGGGAGGTGACAAC |
| **Glucagon** | Forward | CGTAAAAAGCAGATGAGCAAAGTG |
|  | Reverse | GAACAGGTGTAGACAGAGGGAGTCC |
