# Supplementary material for: Repressing miR-23a promotes the transdifferentiation of pancreatic α cells to β cells via negatively regulating the expression of SDF-1α
Source: PLoS One. 2024 Mar 22;19(3):e0299821. doi: 10.1371/journal.pone.0299821 (PMC10959391; doi:10.1371/journal.pone.0299821)
Supplement: Attachment — Submitted filename: Comments Plos One_.docx [file pone.0299821.s005.docx]

The articletitled "Repressing miR-23a promotes the pancreatic α-to-β cells transdifferentiation via SDF-1α" authored by Lang et al. underscores the potential significance of miR-23a as a promising therapeutic target in the context of regenerating pancreatic β cells from α cells. This research aims to enhance insulin production, ultimately offering a novel avenue for treating Type 1 Diabetes (T1D). Before considering this article for publication in the journal, certain issues require attention and resolution, as outlined below.

1. The authors mentioned that mice were considered to have Type 1 Diabetes (T1D) when their blood glucose exceeded 200 mg/dl. However, it is essential to clarify whether this measurement is based on fasting or random blood glucose levels.
2. The authors subjected primary islets to very high glucose levels for an extended period. It would be helpful to provide a rationale for this choice of duration and glucose concentration in the study.
3. The TEM images of exosomes isolated from Min6 cells in Fig. 2A appear to be of poor quality. It is advisable to replace these images with higher-quality representations. Also, please include TEM images of the pancreas or the islets in the treated and untreated groups.
4. In the Oral Glucose Tolerance Test (OGTT) experiment, the blood glucose level at 0 minutes (after 14 hours of fasting) was within the normal range, not in the diabetic range. This discrepancy should be explained in the article.
5. Please provide the beta cell mass values for both the untreated and treated groups of mice, as this information is crucial for understanding the impact of the treatment.
6. How would the treatment affect the diabetes progression it was carried out earlier in the mice, for example in the prediabetes stages? Please comment on this or include data if already collected.
7. While insulin levels increased, and the mice showed improved glucose tolerance upon miR23a treatment, it is important to disclose whether there were any changes in body weight within the treated group of mice.
8. Specify the fold change values for dedifferentiation markers such as Aldh1, Neurog3, and any others mentioned in the study.
9. The authors should examine MafA, Pdx1, Neurod1, Urocortin and Ero1-beta in the alpha cells that are transdifferentiated to beta cells. This would add emphasis to the beta cell identity in after α-to-β cell transdifferentiation. On a related note, XBP1, which is linked to Unfolded Protein Response (UPR) events in cells, has been suggested to maintain beta cell identity and function under metabolic stress (in diabetes). The authors should consider analyzing the status/expression of XBP1 in their models in this study.
10. Please include a brief discussion on epigenetic factors that may influence α-to-β cell transdifferentiation.
11. The Immunohistochemistry (IHC) figure for insulin and glucagon are not convincing, as the images for insulin and glucagon appear to be from different pancreatic sections or islets. Please provide accurate and representative immunofluorescence images.
12. In Fig. 2D, where levels of insulin and glucagon are shown using ELISA assays, it would be better to also present this data using Western blot analysis for a more comprehensive assessment. Good
